# An analysis of humanitarian and health aid alignment over a decade (2011–2019) of the Syrian conflict

**DOI:** 10.1186/s13031-022-00495-5

**Published:** 2022-11-09

**Authors:** Munzer Alkhalil, Maher Alaref, Hala Mkhallalati, Zedoun Alzoubi, Abdulkarim Ekzayez

**Affiliations:** 1 Research for Health System Strengthening in Northern Syria (R4HSSS), Union for Medical and Relief Organizations, Gaziantep, Turkey; 2 Syria Public Health Network, London, UK; 3 MIAD, Paris, France; 4 Strategic Research Center SRC, Gaziantep, Turkey; 5 Syria Research Group (SyRG), Co-hosted By the London School of Hygiene and Tropical Medicine and Saw Swee Hock School of Public Health, London, UK; 6 Research for Health System Strengthening in Northern Syria (R4HSSS), The Centre for Conflict and Health Research (CCHR), King’s College London, London, UK; 7 Nottingham, UK

**Keywords:** Aid alignment, Aid effectiveness, Humanitarian aid, Health aid, 2005 Paris Declaration, Humanitarian crisis, Conflict, Syria

## Abstract

**Background:**

The 11 years of the devastating conflict in Syria resulted in more than 874,000 deaths, and in more than thirteen million refugees and internally displaced people (UNHCR, Syrian refugee crisis: aid, statistics and news, USA for UNHCR, Washington, 2020; Alhiraki et al. in BMJ Glob Health 7:e008624, 2022). The health system was severely affected and has become aid dependent. This study examines aid alignment over a decade of the Syrian crisis from 2011 to 2019.

**Methods:**

Aid alignment involves donors using national systems and institutional structures to manage their aid to recipient governments and aligning their aid policies with development priorities and strategies defined by the partner countries (ROSA Newsletter, Moving towards increased aid alignment in the food and nutrition security sector, 2013. Available from: http://oxfamilibrary.openrepository.com/oxfam/bitstream/10546/141974/1/). Aid alignment was explored as part of the 2005 Paris Declaration Framework on aid effectiveness. Based on OECD’s survey on monitoring the Paris Declaration (OECD, Harmonisation, alignment, results: report on progress, challenges and opportunities, OECD, Paris, 2005; OECD, Survey on harmonisation and alignment of donor practices, OECD, 2006. Available from: https://www.oecd-ilibrary.org/development/survey-on-harmonisation-and-alignment-of-donor-practices_journal_dev-v6-sup1-en) and based on a proposed methodology to assess aid effectiveness by Burall and Roodman (Developing a methodology for assessing aid effectiveness: an options paper, Overseas Development Institute, 2007. Available from: www.odi.org.ukhttp://www.cgdev.org), we designed a sequential mixed methodology to address two main indicators: alignment with national strategies and local procedures, and aid delivery through local systems. The quantitative part investigated the financial alignment of aid using financial data trackers, such as creditor reporting system and the UN-OCHA financial tracking system, and the relevant humanitarian needs estimations by the humanitarian assistance response plans, humanitarian response plans, and humanitarian needs overviews. The qualitative part relied on four focus groups discussions and four key informants interviews with key policy makers, experts and practitioners involved in the humanitarian and health response in Syria, with the aim of interpreting the quantitative findings.

**Results:**

While the study found an improvement in aid budget alignment with local procedures in Syria from 34% in 2012 to 86% in 2019, we found limited alignment with local strategies. Our qualitative findings pose doubts in the ability of the various data sources of humanitarian needs in Syria to reflect the actual realities, especially before 2014, due to lack of comprehensive local engagement and data systems by then. Therefore, even if the humanitarian budgets seemed to be aligned with the national procedures, the national plans did not seem to align with the actual realities, let alone the increase in the financing deficit over the years of the conflict. The reliance of humanitarian and health aid on governmental structures, as a main recipient, in Syria was much lower than other developing and fragile countries. This is mainly due to the nature of the Syrian conflict where the government is a party to the conflict. Donors were found to have invested poorly in advancing national and sub-national planning in Syria due to donors’ over reliance on the UN-led humanitarian system which struggles in armed conflict settings. As a result, we found a disconnection between field realities, national planning, and humanitarian aid.

**Conclusion:**

In light of the dreadful humanitarian crisis in Syria, there has been an adverse aid alignment. Considering the chronicity of the conflict, there is an urgent need to improve aid alignment through more investment in local planning at district or governorate levels. This is especially important to navigate through conflict sensitivities while responding to local needs and initiating local developments. These approaches, combined with adopting health sector-wide approach, could contribute to the humanitarian-development-peace nexus in Syria, which in turn can contribute to a better aid alignment and aid effectiveness.

## Introduction

Armed conflict settings are often very aid-dependent, and there are copious amounts of aid received by countries affected by conflicts. While there have been several studies tracking levels of aid to conflict-affected countries [7], there has been a dearth of research on aid effectiveness in these contexts.

Aid effectiveness can be defined, as per the World Bank, as “the impact that aid has in reducing poverty and inequality, increasing growth, building capacity, and accelerating achievement of the development goals set by the international community” [8]. Aid effectiveness thus expresses the success or failure of aid to developing countries. In conflict settings, although donors try to play a neutral role, the impact of aid is not neutral and becomes part of the conflict mechanisms [9].

Donors have made efforts to maximize aid impact and deepen development cooperation. These efforts have resulted in formulating fundamental principles and measures for aid effectiveness through four high-level forums in Rome, Paris, Accra and Busan in 2003, 2005, 2008, and 2011 respectively [10]. However, during the past decade, donors have not given sufficient attention to aid effectiveness, which might have limited the positive impact that aid can have in recipient countries [11]. For humanitarian aid, a group of donors endorsed the Principles and Good Practice of Good Humanitarian Donorship (GHD) in Stockholm in 2003 to enhance donor effectiveness, coherence, accountability to beneficiaries, domestic constituencies, and implementing organisations [12].

In the literature, there is an argument that the "aid principles" landscape has become significantly crowded recently, with the 2005 Paris Declaration, the Good Humanitarian Donorship agenda and the Fragile States Principles as examples of several different initiatives. However, each of these frameworks is designed to serve specific type of aid; humanitarian, early recovery, stabilisation, or development. It can be argued that many contexts are more complicated than these categories of aid. Additionally, humanitarian actions do not occur in isolation within humanitarian crises but as part of a vast sphere of international interests, especially in conflict settings. As a result, donors should adopt different principles and frameworks, sometimes in the same context [13].

In the Syrian context, there is a transition from "emergency" to "early recovery" after more than 11 years of the conflict [14]. Donors face a real challenge regarding the need to provide humanitarian, transitional and development aid to meet the requirement of the triple nexus (Humanitarian, Development, Peacebuilding). Therefore, we chose the Paris declaration as a framework to assess aid effectiveness in Syria to be able to investigate both humanitarian and development aid with a focus on a possible transition in aid from humanitarian to development in the last few years of the Syrian conflict.

According to the 2005 Paris declaration, there are five main principles for aid effectiveness: ownership, alignment, harmonization, managing for results and mutual accountability [15]. Our study focuses on examining aid alignment in the Syrian conflict between 2011 and 2019.

To achieve the principle of alignment in the Paris Declaration, donors must “base their overall support on partner countries’ national development strategies, institutions and procedures” [15]. Accordingly, the OECD developed few indicators to assess the various elements of aid effectiveness. For aid alignment, two indicators were identified: Alignment with local development strategies, including budget support is aligned with local procedures; and project support is delivered through local systems. We tried to assess these two indicators in Syria to investigate aid alignment there.

Considering the scarcity of the literature on humanitarian aid effectiveness in conflict environments, a key question remains unanswered: Is humanitarian aid less likely to achieve the principles of aid alignment in contexts of conflict and violence? And is this linked to the lack of governance and accountability and the absence of governmental structures in such settings? The Syrian case study might help in answering these questions in relation to aid alignment.

### Study setting

The Syrian crisis was described as “the worst humanitarian crisis of the twenty-first century” [16]. From the outbreak of protests in Syria in 2011 until 2020, the conflict seems to have caused about 874,000 excess deaths—directly and indirectly [2], among which about 606,000 people were killed [17]. Of the 22 million pre-conflict population of Syria, more than 13 million became refugees and internally displaced [18]. 98% of people inside Syria live in extreme poverty, as they live on less than $ 1.90 per person per day [19].

**Fig. 1:**
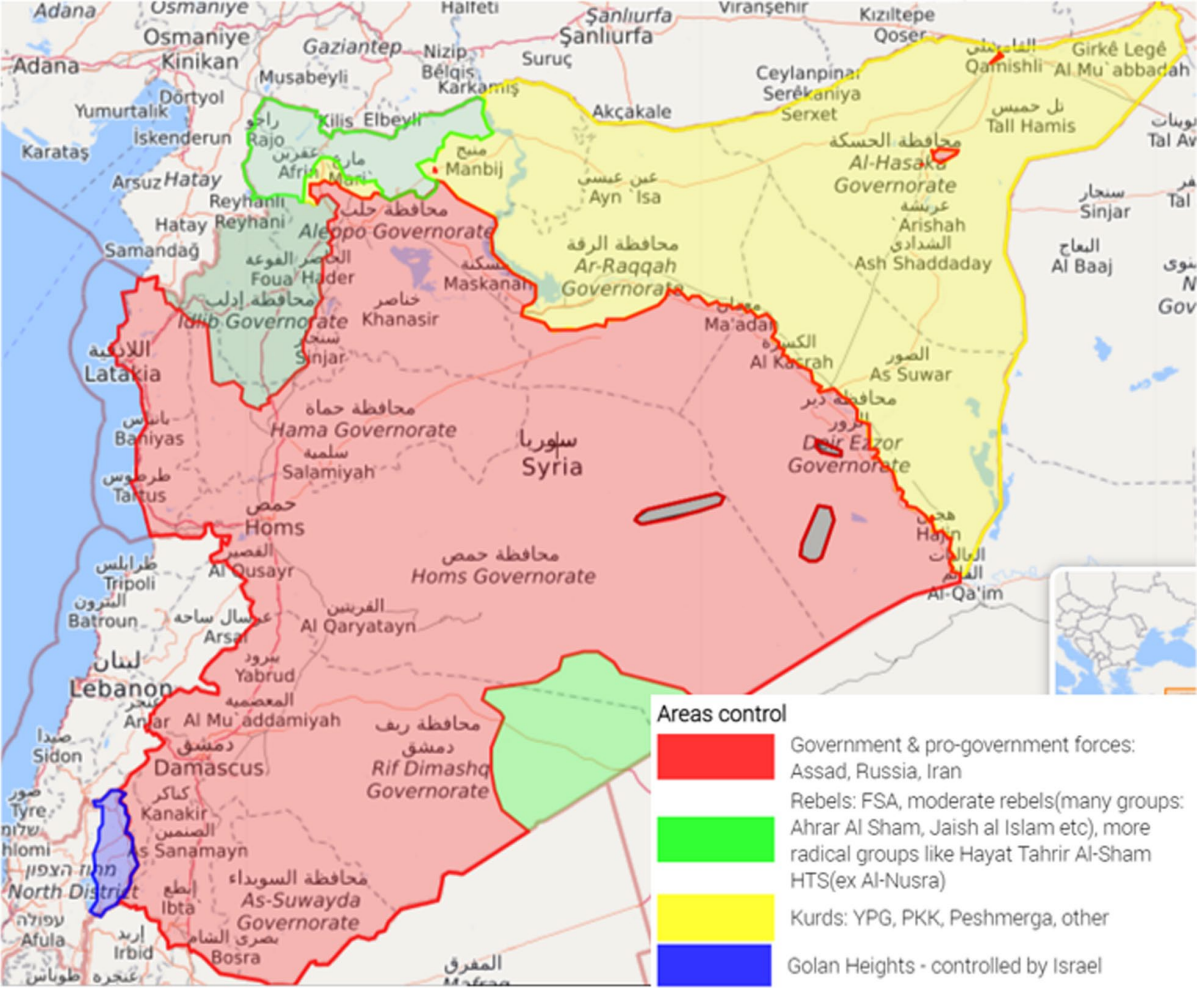
Areas of control in Syria as of Jan 2019—Source: Live Universal Awareness Map (Liveuamap)

The areas of military influence changed dramatically in Syria between 2011 and 2019, and this was accompanied by significant changes at the level of governance and humanitarian and health aid. Figure 1 illustrates three main areas of control as of January 2019.

For simplification, we can distinguish three main areas of control in Syria. (1) The GoS controls central, coastal, and southern areas supported by Russia, Iran, and Shia militias such as Hizabullah. (2) Various opposition forces control areas in northwest Syria in Idlib and Aleppo governorates supported mainly by Turkey. In these areas, two governments emerged; the Syrian Interim Government, which as formed by the National Coalition of Syrian Revolutionary and Opposition Forces in 2013 and operates in Turkish controlled areas in northern Aleppo [20–22], and the Syrian Salvation Government (SSG), which was formed by Hayat Tahrir Al-Sham (HTS) and operated in Idlib governorate [23], (3) The Autonomous Administration of North and East Syria (AANES) controls areas in northwest Syria supported by the USA and other international powers [24, 25].

The Syrian crisis necessitated a large scale humanitarian response which started in 2011 through local and international NGOs. In 2014, the UN Security Council adopted Resolution 2165 allowing UN agencies to provide aid into Syria through four border crossings to facilitate aid access to areas not under the control of the Syrian government: Bab al-Salam and Bab al-Hawa to deliver aid into northwest Syria, Al Yarubiyah to deliver aid into northeast Syria, and Al-Ramtha to deliver aid into southern Syria [26]. This resulted in three distinctive humanitarian hubs in Syria; one in Damascus delivering aid to areas under the control of the Government of Syria (GoS), another in Gaziantep/Turkey delivering aid to opposition and Kurdish forces controlled areas in northern Syria, and a third in Jordan delivering aid to opposition controlled areas in southern Syria. To coordinate these three humanitarian hubs the Whole of Syria approach (WoS) was established in Amman/Jordan in 2015 [27]. The coordination mechanisms of the humanitarian response are run by the UN Office for the Coordination of Humanitarian Affairs (OCHA), which is responsible for leading the Humanitarian Response Plans (HRPs) and Humanitarian Needs Overviews (HNOs) and documenting the development process [28]. However, the UN-led cross border aid in southern and in northeast Syria ended in 2019 after the Russian and Chinese vetoed the extension of the UNSCR to deliver aid to these areas cross the border. Bab al-Hawa border was the only exception and it remains open, until the writing of this paper in Sep 2022, to deliver aid to opposition controlled areas in northwest Syria [29]. Although this veto affected the UN led cross border response in northeast Syria, another humanitarian hub emerged there coordinated by the NES NGOs Forum. The NGOs Forum was founded by non-governmental organizations (NGOs) that operate in northeast Syria as a coordination platform to lead the cross-border and the local humanitarian response in northeast Syria. The NGO Forum manages various technical working groups, and it has loose links with the WoS approach in Amman from the one hand, and with the local authorities in northeast Syria from the other hand.

Although the Syrian government has been accused of several war crimes including the use of chemical weapons against its own people [30, 31], it is still considered as the legitimate Syrian government at the United Nations. Most of the aid financial trackers, such as the OECD’s Creditor Reporting System (CRS), therefore, recognise the Syrian government and not the other de facto governments in the different areas of control [32]. It is crucial for us, therefore, not to limit our analysis to financial data from such trackers due to the bias of relying only on structures and plans of the Syrian government. We distinguish, therefore, in the Syrian context between the “national” level—which refers to structures and processes led by the internationally recognised government in Damascus; and the “sub-national” level, which concerns structures and processes on the level of each area of control; and the “local” level, which concerns structures and processes at the levels of governorates, districts, and communities. This is to accommodate the complex geopolitics of the Syrian conflict where “national” level can be tricky to define in the different areas of control, and where the GoS, the internationally recognised government, is a party to the conflict. We considered thus the different actors that should be involved in assessing needs, developing response plans and budgets, and leading implementation and evaluation at each level.

According to the UN-OCHA, the top ten humanitarian donors for Syria in 2019 were: the USA, Germany, UK, EU, Canada, Norway, Denmark, Japan, France, and Sweden, respectively. And the top funded sectors in 2019 both inside and outside the response plan/appeal were: food security, health, education, water sanitation hygiene, emergency shelter and non-food items (NFI), and protection, respectively [33].

## Methods

### Study design

The study used a sequential mixed methodology to explore aid alignment in Syria between 2011 and 2019. The study design was informed by the OECD’s survey on monitoring the Paris Declaration [4, 5], and by a proposed methodology to assess aid effectiveness by Burall and Roodman in 2007 [6]. The study tried to assess aid alignment through two main indicators: (1) alignment with local strategies and local procedures, and (2) aid delivery through local systems.

The quantitative part investigated the two indicators using financial data trackers, such as Creditor Reporting System (CRS) and the UN-OCHA financial tracking system (FTS), alongside with using the relevant humanitarian needs estimations by the Humanitarian Assistance Response Plans (HARPs), Humanitarian Response Plans (HRPs), and Humanitarian Needs Overviews (HNOs). The qualitative part was aimed at filling gaps and interpreting the quantitative findings relying on four Focus Groups Discussions (FGDs) with experts and practitioners working for NGOs, INGOs, local health authorities, and health facilities inside Syria all of them are involved in the humanitarian and/or health response in northwest of Syria, and some of them are also involved in northeast Syria. Four Key Informant Interviews (KIIs) with key policy makers and donors involved in the humanitarian and health response in the delivering aid to the various areas of control was then used to validate our findings and form our recommendations.

### Quantitative analysis

#### Data sources

Data on humanitarian and health aid were extracted from the OECD’s Creditor Reporting System (CRS) [32], and the OCHA’s Financial Tracking System (FTS) [34]. Despite of some limitations of the CRS and the FTS systems, these databases combined provide a reliable source for tracking health and humanitarian aid for conflict-affected countries. These datasets allow for analysis of different aid activities, multilateral and philanthropic donors, country donors and recipients, purpose, policies, and years [35, 36]. The CRS data used in this analysis are based on the 31 April 2021 update [32], and the dataset was downloaded on 15 August 2021. The FTS data were downloaded to an excel sheet on 17 August 2021. The timeframe of this quantitative analysis was selected to be from 2011, being the start year of the Syrian crisis, to 2019, the last year available on the CRS database when we started the study.

There are some differences between the CRS and the FTS datasets due to the different focus of each dataset; with the former being focused on development aid, and the latter being focused on humanitarian aid. The CRS data, therefore, are more comprehensive and offer more details at donors’ and recipients’ levels, including different sectors and reporting values in both constant and current US dollars. In comparison, the FTS data provide continuously updated monitoring of funding progress against the Humanitarian Response Plans and appeal requirements [37].

In this analysis, the term ‘aid’ includes ‘Official Development Assistance’, ‘ODA grants’, ‘ODA loans’, and ‘Private Development Finance’. Aid excludes ‘Equity Investment’ and ‘Other Official Flows’ [32, 38, 39]. Data were extracted based on gross disbursements rather than commitments, because we were looking at “the actual international transfer of financial aid” rather than resources, or goods or services valued at the cost to the donor [40].

To analyse aid trends over the study period, we relied on the constant 2019 US dollar value rather than the current dollar value to account for changes in exchange rates and inflation. The Development Assistance Committee (DAC) deflator converts the amounts back to the value they held in a specific year; in our example, 2019 [41, 42]. The aid database includes the bilateral ODA of the DAC members, and it excludes their contributions to the regular budgets of multilateral institutions when accounting for bilateral aid [41].

We excluded the Turkish humanitarian donor from our analysis due to a classification error in their reporting on the CRS system. The Turkish donor reports the expenses on Syrian refugees in Turkey as an ODA grant, although these expenses did not reach the recipient country—in this case Syria.

Data on humanitarian needs and local strategies were extracted from the OCHA database FTS. The FTS database includes inputs from the OCHA’s humanitarian and response plans (HRPs) [43] and the OCHA’ people in need’ (PIN) projection [44]; both of these sources combined can be considered a reliable reflection of national and sub-national strategies and local needs. Alignment with needs and strategies was assessed by determining proportions of funding aligned with OCHA-approved plans from the total funding reported to the FTS. Also, annual plans coverage ratios were calculated during the study period.

Data on the use of local systems were extracted from the CRS database. The Paris Declaration recommended that donors use ‘recipient countries’ systems and policies to promote development goals [15]. The percentage of health and humanitarian aid that flows through government channels was calculated for each year. These ratios were compared with the global ratios of all fragile and developing countries receiving aid.

#### Quantitative variables

Relying on the above-mentioned databases, we identified several variables to represent donors’ disbursements, humanitarian local strategies and procedures, and the use of local systems. The following table summarises the variables used in the quantitative analysis (Table 1).

**Table 1:** List of variables included in the quantitative analysis

Domain	Variable	Source	Comments
Donors’ disbursements	Health aid	CRS	This variable was a sum of the following variables*: (1) Health General (121) (2) Basic Health (122) (3) Non-communicable diseases (123) (4) Population Policies/Programmes and Reproductive Health (130)
	Humanitarian aid	CRS	This variable was a sum of the following variables*: (1) Emergency response (720) (2) Reconstruction, relief, and rehabilitation (730) (3) Disaster prevention and preparedness (740)
Humanitarian local strategies and procedures	Percentage of humanitarian plan coverage	FTS	The FTS calculates this variable based on the HRP and the reported funding
	Percentage of humanitarian funding through plans	FTS	The FTS calculates this variable based on the HRP and the reported funding
	Number of people in need of humanitarian assistance	PIN	This variable was extracted from the “People In Needs” data from OCHA
Use of local systems and channels	The percentage of health aid through a recipient government	CRS	These two variables were extracted from the CRS data where “recipient government” is named as the “channel reported”
	The percentage of humanitarian aid through a recipient government	CRS	

* The CRS database has a coding system that identify the purpose of the funding. Data on the destination sector are recorded using 5-digit purpose codes; it is a compulsory classification due to the specific areas that aid tends to support in the recipient’s countries. Thus, aid was allocated to the most suitable code available within each sector [45]. Health aid was outlined by DAC 5 CODE 120: I.2. to 130: I.3, which includes Health General (121), Basic Health (122), Non-communicable diseases (123), and Population Policies/Programmes & Reproductive Health (130). Therefore, health aid represented just non-humanitarian health. So, there is no duplication with humanitarian aid. Humanitarian aid was outlined by DAC 5 CODE with 700: III, which includes: emergency response (720), reconstruction, relief, and rehabilitation (730) and disaster prevention and preparedness (740). These sub-categories have many health-related aspects in emergencies and humanitarian aspects. Nevertheless, they do not intersect with non-humanitarian health sub-categories in the health sector

### Qualitative analysis

The financial data were insufficient to understand aid alignment in Syria. We complemented the quantitative analysis with four Focus Group Discussions (FGDs) and four semi-structured interviews with experts, humanitarian practitioners, and public sector officials to fill gaps and interpret the quantitative findings. This was crucial because the quantitative data were not an accurate reflection of what is happening on the ground. This might be related to incompleteness and misclassifications in financial reporting, but more importantly it is related to the conflict sensitivities where the GoS is recognised in such databases as the only representative of national planning, strategies, and local systems.

We used purposive sampling followed by snowballing sampling approaches to identify the FGDs participants. The research team invited 31 humanitarian workers in senior positions from medical NGOs and INGOs, local authorities, technical entities, and the Gaziantep’s health cluster to take part in the FGDs in Turkey—Mersin in August 2021. 25 out of the 31 invitees attended the FGDs, and 88% of the participants were from a medical background with a vast experience in humanitarian and health programs. Four FGDs were conducted with an average of 12 participants in each one. The discussions were conducted in Arabic, with one moderator and one note taker for each group. The FGDs were not recorded based on the participants’ preference.

The FGDs were followed by four semi-structured interviews with representatives of 4 leading donors in Syria. The interviews aimed at validating our findings and initiating discussions related to policy implications. The feedback from these key informants aligned strongly with areas discussed in the FGDs. The KIIs were conducted in English and they were recorded. They were later transcribed and anonymized using a unique identifier for each participant. Following a thematic analysis approach, data from the FGDs and the interviews was extracted and categorized into different themes.

## Results

### Alignment with national needs and strategies

#### Overall improvements in aid budget alignment with local needs and strategies

**Fig. 2:**
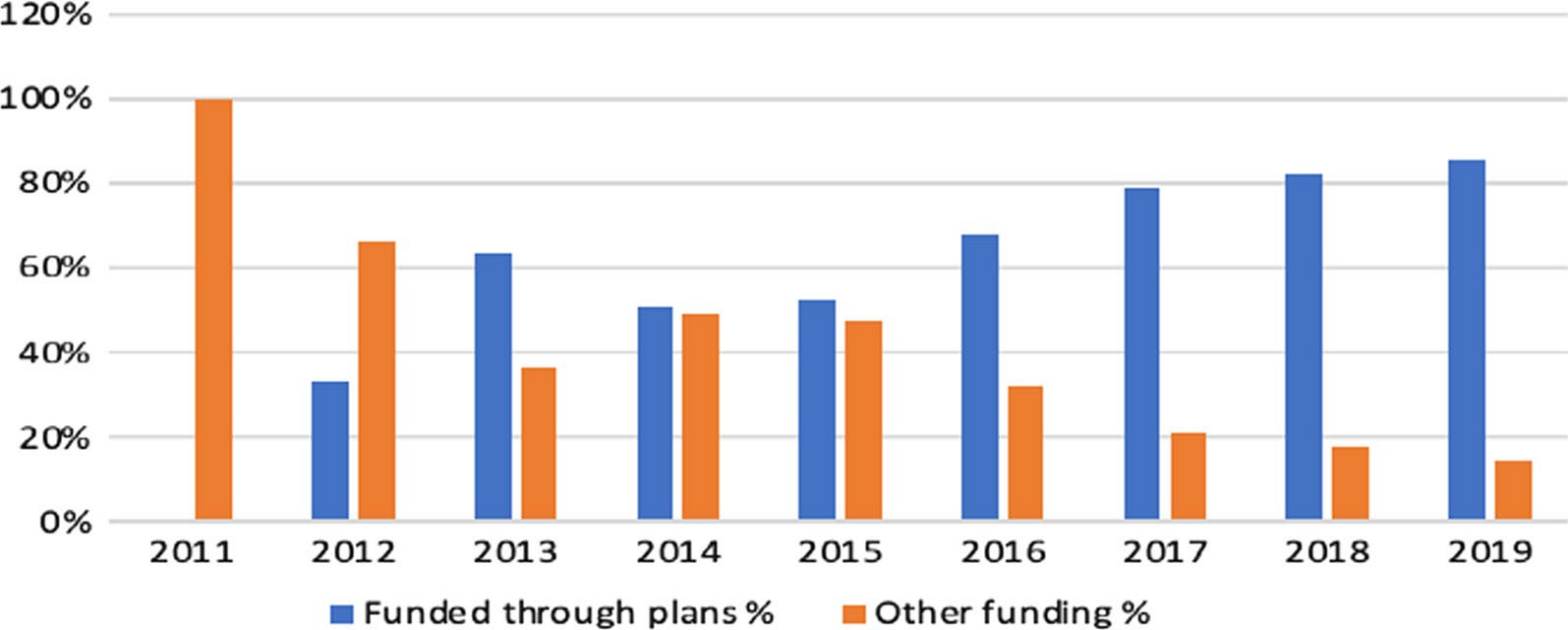
Humanitarian funding for Syria 2011–2019 (FTS)

In 2011, 100% of the humanitarian funding was spent without a plan. Between 2012 and 2019, funding without any plan decreased from 66% in 2012 to 14% in 2019. In other words, plan-aligned funding increased from 34% in 2012 to 86% in 2019. Overall, there was an increase in alignment with national needs and strategies over the study period (Fig. 2). There are no essential differences between funding through and outside the plans regarding the selected sectors. However, the funding outside the plans depends on direct intervention by donors based on their own plans or agendas. In contrast, funding through the plans depends mainly on the HRPs.

**Fig. 3:**
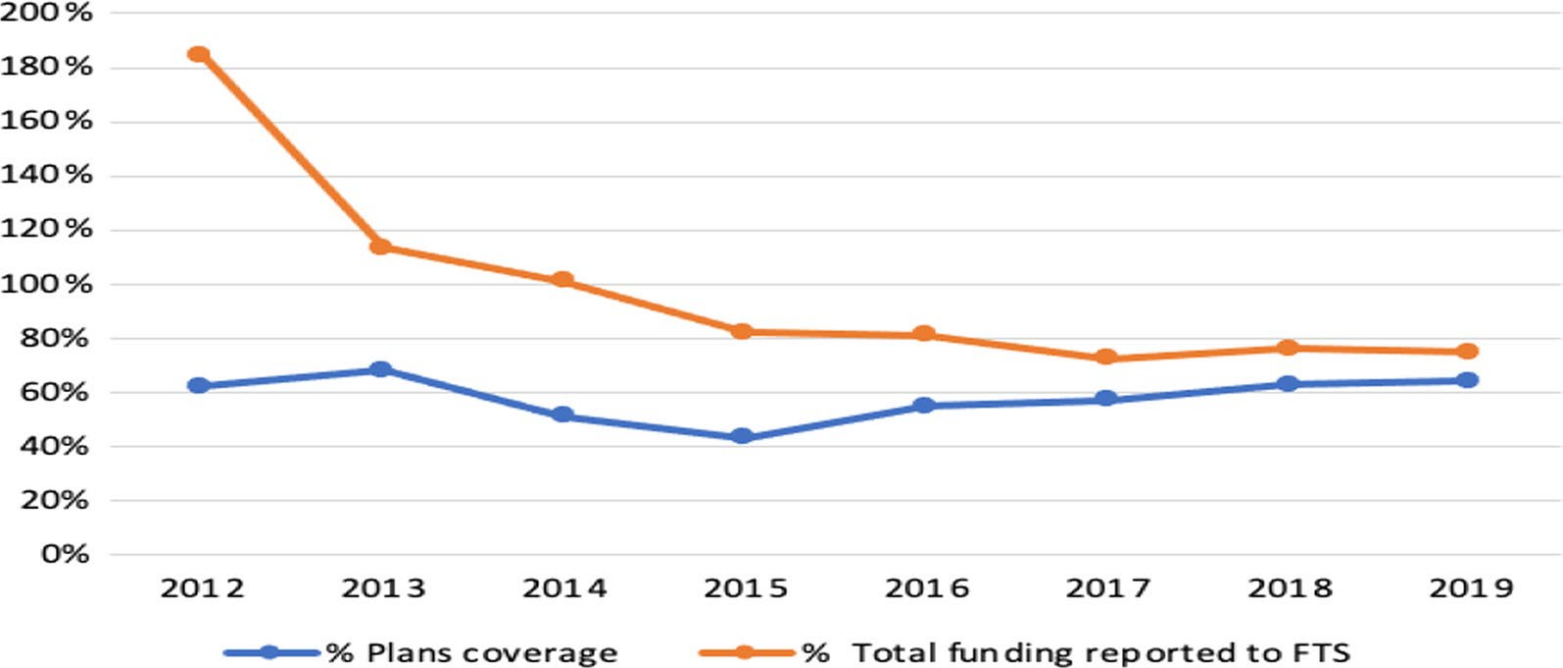
Comparison between humanitarian funding and plans coverage in Syria 2011–2019 (FTS)

Figure 3 illustrates that the percentage of the total funding reported to FTS decreased steadily during the study period compared to the required fund; from almost 184% in 2012 to 75% in 2019. However, plans coverage started at around 60% in 2012, peaked in 2013 at 68%, and bottomed out in 2015 at around 53%, eventually settling on a ratio similar to the starting ratio of about 64%.

**Fig. 4:**
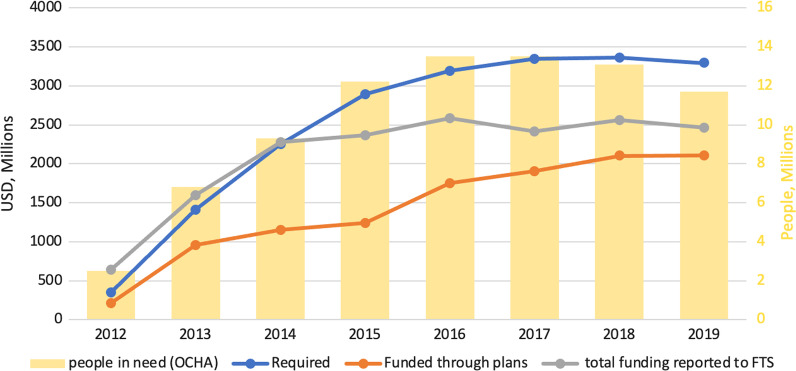
Humanitarian aid trends VS People in need in Syria 2011–2019 (FTS)

It can be seen in Fig. 4 that while aid funded through plans and total funding reported to FTS increased over time, there was a growing gap between the required funding and the total funding after 2014, and between the required funding and the funding through plans over the study period. However, there was an alignment between the required funding and the number of people in need.

#### Available data might not reflect the actual realities on the ground

The FGDs and the KIIs focused on how the HRP’s and the HNO’s methods were developed under the leadership of the UNOCHA. Participants confirmed that the available data do not reflect reality before 2014, which is the year when the cross-border UN-led humanitarian response started in opposition held areas. Since then, the humanitarian cluster system, led by the OCHA, has been responsible for leading the Humanitarian Response Plans (HRPs) and Humanitarian Needs Overviews (HNOs) and documenting the development process. Therefore, reliable data about humanitarian needs and appealed funds were unavailable for all parts of the country before 2014.

The participants argued that even after 2015, representative data on aid budget alignment with local plans might be limited due to an absence of national bodies to support the development of nation-wide and needs-based response strategies. While the cluster system involves local humanitarian actors in developing response strategies, the engagement of local governance structures in northwest and in northeast Syria in such processes was almost absent. These indicators, therefore, might poorly represent the actual reality on the ground, especially in northwest and in northeast Syria. So, the actual concept of local needs and strategies was not reflected in the mentioned process. For example, several participants mentioned the case of the Strategic Advisory Group, which was established by the Whole of Syria coordination mechanism as part of the health clusters in the various humanitarian hubs. This advisory group in Gaziantep health cluster started in 2016 to engage all local actors—including local governance structures—in needs assessment and response strategies. However, this advisory group has been deactivated in 2018, and accordingly the governance bodies in northwest Syria have since not been involved in needs assessments and in developing response strategies. This deactivation remarkably impacted the local perspectives of needs and response strategies in the development process of the HNO and the HRP. The participants argued that the subsequent HRPs, in 2019 and 2020, poorly represented the actual needs and the sub-national response strategies in northwest and in northeast Syria. The 2021 HRP had not been released yet at the time of the workshop in Mersin in August 2021.

Another challenge that might hindered the completeness and the representation of data is the conflict complex geopolitics and the accompanied complexity in the humanitarian structures to navigate through these geopolitics. One participant mentioned that the HNO is usually affected by the political and the military influences in the various areas of control. For example, the GoS is heavily involved in needs assessment in its areas of control. There is precedent evidence that suggests the manipulation of humanitarian aid by the Assad regime [46, 47]. The participants argued that the GoS always submits questionable and mostly manipulated data to the HNO and the HRP. Since 2019, the GoS started to base their data on the overall number of people in governmental controlled areas rather than on the actual number of people in needs. Additionally, the GoS estimation of vulnerability does not take into account the available infrastructure and resources in its areas of control. On the other hand, there is an absence in the participation of sub-national governmental authorities in both northwest and northeast Syria.

### Alignment: aid channelled through governmental bodies

#### Very limited use of governmental channels for both humanitarian and health aid in Syria

**Fig. 5:**
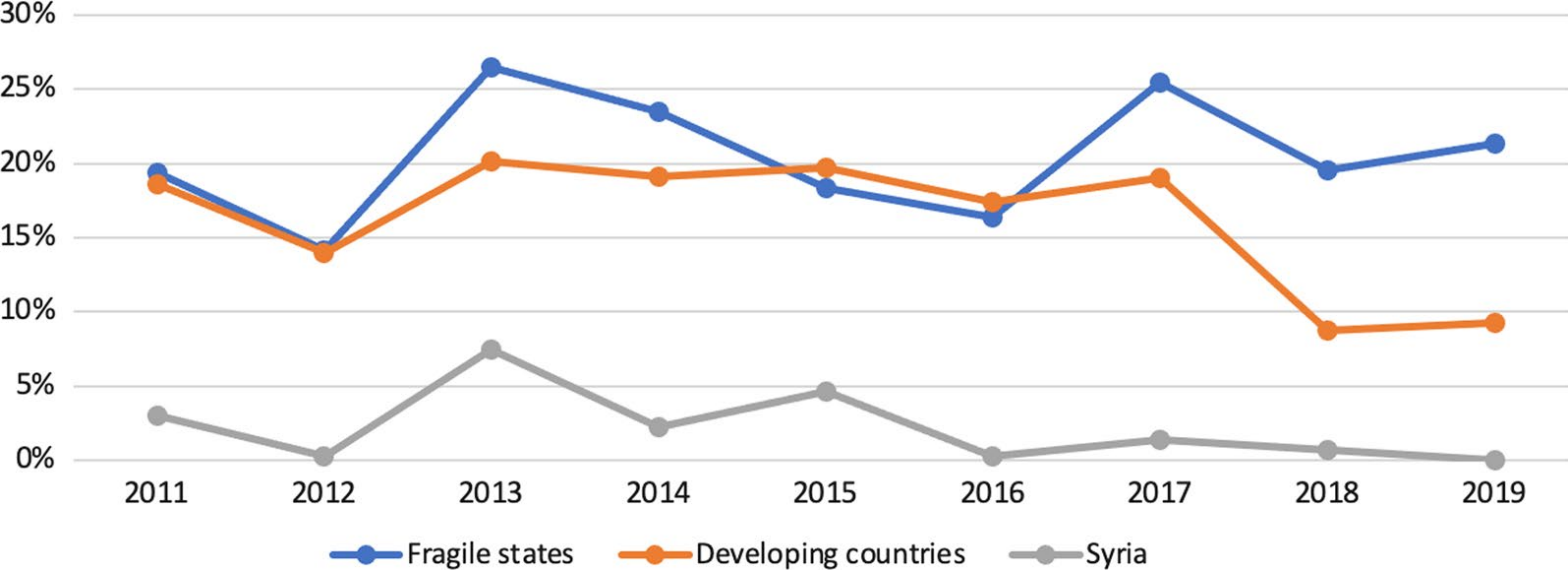
Health aid through a recipient government 2011–2019 (CRS)

During the period of our study, we found a tendency for health donors to work through a recipient government in both fragile states and developing countries (Fig. 5). The situation in Syria was found to be different, as the percentage of health funding that passes through the recipient government as a first channel is much lower than that of fragile and developing countries in all years of the study. This is, according to the FGDs and the KIIs, because the GoS was receiving very limited non-humanitarian health funding over the 10 years of the conflict. However, we noted an increased use of the government’s channel in 2013 and 2015 in Syria.

**Fig. 6:**
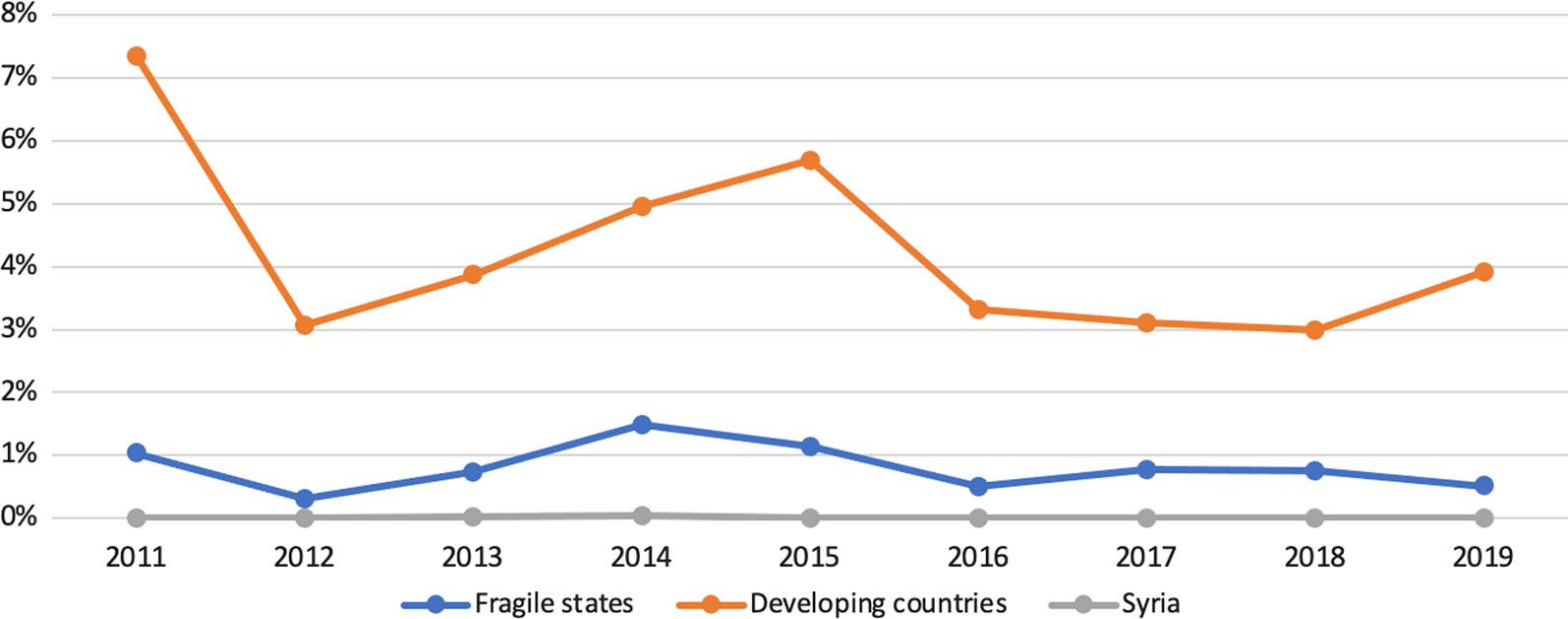
Humanitarian aid through a recipient government 2011–2019 (CRS)

In contrast to health aid, donors were found to use channels of recipient governments for humanitarian funding in developing countries much more than in fragile states (Fig. 6). This might be due to political and military complexity in fragile settings where national governments are not perceived as capable of receiving humanitarian funding. For the Syrian context, the percentages of humanitarian funding going through the GoS are close to zero during the 10 years of the conflict. According to the FGDs and the KIIs, these findings are in line with the humanitarian principles of neutrality and impartiality considering that GoS is a party to the conflict.

#### The need to re-define the use of local systems by humanitarian and health aid

The CRS and the FTS databases recognise official governmental bodies only when calculating the percentage of aid going through local systems. The participants in the FGDs argued that this classification should be redefined, especially in conflict settings such as Syria. In conflict settings, the humanitarian principles prevent humanitarian donors and actors from engaging directly with parties to the conflict. In the case of Syria, the GoS is one party to the conflict, which was accused with many war crimes including the use of chemical weapons. The participants, thus, suggested that the use of local systems should be extended to other local actors and grassroot governance structures that represent sub-national levels and local communities that are not parties to the conflict.

Additionally, the figures on aid going through the Syria government channels do not represent all aid going through local systems and channels, as the Syrian government does not control all of the Syrian territories. For example, the participants reported that some funding was going through sub-national governmental bodies, such as the Syrian Interim Government in northwest Syria and the Autonomous Administration of North and East Syria; this funding was not reported as going through local systems on neither of the financial tracking databases. There is no information in the CRS system on whether some Syria related funding passed through governmental structures other than the structures and the channels of the GoS.

#### The manipulation of aid by the Assad regime

Although the reviewed data suggest that the use of the GoS channels has been very limited, the participants of the FGDs argued that this data might be biased. They argued that the data show the percentages of funds passed through the official governmental bodies of the GoS as a first channel; the data, however, do not consider other channels that are closely affiliated with the GoS, such as the Syrian Arab Red Crescent, Al Bostan charity, and the Syria Trust for Development. These NGOs received significant funding as a first or a second channel, and their response strategies are completely aligned with the GoS plans.

Furthermore, the participants discussed the balance between the use of local systems and the possibility of empowering parties to the conflict. While the Paris declaration has an embedded aim that humanitarian responses should contribute to the recipient country’s resilience, this assumption might be tricky to implement in chronic conflict settings where national governments can be parties to the conflict.

The participants recalled several incidents when the GoS manipulated humanitarian funding for its own interests, which might have contributed to strengthening its political and military position. This opinion is in line with many reports that mentioned impeding the supply of UN aid to some besieged areas by the GoS, and the flow of UN aid funds to the GoS through NGOs established by relatives of the Syrian President, such as the Syria Trust for Development—which is headed by Asmaa Al Assad, and Al-Bustan Association—which is run by Rami Makhlof who is a member of the Assda’s family [46, 47].

There should be, therefore, other mechanisms to work with local systems in Syria to increase the sustainability of humanitarian aid without empowering parties to the conflict.

## Conclusion and discussion

There have been very limited studies that assessed aid alignment in conflict settings. A review in 2004 for international aid in fragile settings suggested that the incentive effect of aid reform in fragile states is highly doubtful [48]. In Southern Sudan, a report in 2018 found poor aid alignment and a need to increase donors’ cooperation and strengthen the government’s financial system [36]. Another study in Afghanistan in 2012 found that aid is often ineffective, fuels nepotism and corruption in fragile states, especially in conflict, and is rarely an effective development tool [49].

This study contributed to the efforts of evaluating progress towards better aid effectiveness through assessing aid alignment in the Syrian conflict. The official financial trackers, such as the CRS and the FTS, indicate an improvement in aid alignment in Syria between 2011 and 2019 from 34% in 2012 to 86% in 2019—compared to the UNOCHA’s humanitarian and response plans (HRPs). However, our study suggests that this improvement might only poorly reflect the actual realities on the ground. Issues such as data completeness and representation, conflict geopolitics and data transparency, manipulation and politicisation of aid, and the way national and local systems and channels are defined, are all challenges to be considered when assessing aid alignment learning from the Syrian context. Our study thus proposes a key question: how can aid alignment be improved in a context where the internationally recognised government is accused of war crimes?

Humanitarian funding increased significantly in Syria throughout the 9 years of the study, in line with the increase in humanitarian needs. In parallel, the availability and the quality of needs assessments and planning processes also improved significantly. In the early years of the conflict, there was no official UN led response covering the whole country. It was not until July 2014, that the UN agencies were allowed to lead the cross border aid to areas out of the control of the GoS. The humanitarian response planning, thus, was very limited before 2014. This explains why the humanitarian funding in 2012 and 2013 was more than the required plans. In 2014, the humanitarian funding was almost equal to the response plans. Later, the deficit in humanitarian funding increased from 2015 onwards. The first consolidated appeal for Syria was launched in 2015 after the UNOCHA led a nation-wide planning process covering the different areas of control, which resulted in the 2015 Syria Strategic Response Plan (SRP) and the 2015 Humanitarian Needs Overview (HNO) [50].

For health funding, which includes both humanitarian and development funding, the gaps in data are more remarkable. There are no overall response plans for the health sector—that covers the whole country—over the study period, neither from the GoS nor from the UNOCHA. Therefore, we were not able to determine to what extent health aid was aligned with the local needs. However, available data showed that health aid had some slight alignment in 2013 and 2015 in Syria, corresponding to the significant peaks in internal displacements in Syria in 2013—with about 3.5 million IDPs [51], and to the launch of the whole of Syria approach in 2015. Another slight improvement in health aid alignment was found in 2017 corresponding to the second significant peak in internal displacements with about 2.9 million new IDPs at that time [51].

The proportion of health and humanitarian aid passed through the structures of the recipient government as the first channel in Syria was much lower than highly fragile and developing countries. Avoiding governmental channels by donors in conflict settings is consistent with the literature, as donors tend to avoid state channels when political commitment is lacking [52]. This has special importance in Syria since the Syrian government is accused of many war crimes, including besiegement, bombardments of civilian areas, and the use of chemical weapons [53–63]. Additionally, there are many reports on the manipulation of aid by the Assad regime [46, 47]. Although many countries, including major donors, imposed unilateral sanctions on the Assad regime [64–66], almost all of these sanctions exclude humanitarian aid [67]. The Assad regime thus focused primarily on humanitarian aid as the only access to international funding. However, avoiding GoS channels should not mean excluding all local channels. There is a variety of local structures, including bottom-up local governance structures and local NGOs, that can act as local systems to receive humanitarian and health aid.

## Recommendations

Avoiding governmental channels while ensuring aid alignment in conflict settings can be very challenging. However, it can be done sometimes through working with other local structures at a much lower level, such as governorate or district level. Aid alignment can also be done in such cases through what is called “shadow alignment”. This means that donors can implement projects based on the government’s vision without allocating resources directly through governmental channels [68]. In Syria, the shadowing can be done by improving the UNOCHA led needs assessments and response planning to involve as many as possible of local actors and bottom-up governance structures in the different areas of control. This is especially important in northwest and northeast Syria where there are well-developed local health bodies that can develop sub-national or district level strategies. This would inevitably contribute to a better aid alignment in Northern Syria. In these two areas, health donors can also use sector-wide approach (SWAp) [69], in contrast to the traditional project approach which is currently in use. This can help strengthening district-level local capabilities within the existing reality of the de facto decentralization in Syria. On the long run, this can contribute to better local leadership and ownership. It can also improve the preparedness for the local channels for resources flow, which in turn paves the way for more balanced local developments.

In GoS controlled areas, donors can negotiate with the GoS to work directly with local authorities on governorate or district levels without going through the central government. This approach can be appropriate to work in the different areas of control in Syria, as working with the central sub-national health authorities represented by the ministries of health of the different governments, carries risks of corruption, politicization and manipulation of aid. Whereas, working at the level of the health directorates or the other health bodies at governance or district levels, can contribute to increasing the participation of local communities, increasing their ownership of development plans, and reducing the politicization of health aid. However, this is a context specific recommendation as avoiding central governments in recipient countries could weaken the governance and accountability structures.

The split of Syria into different areas of control seems to be the de facto reality for the foreseeable future [70, 71]. Considering the active contact lines between these different areas of control and the possible politicisation and militarisation of aid, humanitarian aid should continue to be delivered cross the border into areas out of the GoS control in northwest and in northeast Syria. These cross border humanitarian operations are threatened by the Russian and the Chinese vetoes at the Security Council. In 2019, these vetoes led to the expiration of the humanitarian border crossing into northeast Syria [29]. The humanitarian operations in northwest of Syria are also under continuous threat. Humanitarian donors should work on developing alternative mechanisms for cross-border humanitarian operations to avoid the politicisation of such decisions at the Security Council. This will ensure more aid effectiveness in the various areas of control in Syria.

## Data Availability

All of the data sources used in this study were publicly accessible.
